# Exploratory Association of 12S-HETE with Postprandial Hyperinsulinemia Rather than HOMA-IR in Women with PCOS/PMOS

**DOI:** 10.3390/ijms27219626

**Published:** 2026-10-27

**Authors:** Małgorzata Szczuko, Marta Skórka-Majewicz, Wojciech Żwierełło, Aleksandra Pęcak, Leon Rudak, Urszula Szczuko, Kamila Pokorska-Niewiada

**Affiliations:** 1 Department of Bromatology and Diagnostic Nutrition, Pomeranian Medical University, 70-111 Szczecin, Polandleon.rudak88@gmail.com (L.R.); urszula.szczuko@gmail.com (U.S.); 2 Department of Medical Chemistry, West Pomeranian University of Technology in Szczecin, al. Piastów 17, 70-310 Szczecin, Poland; marta.skorka.majewicz@pum.edu.pl (M.S.-M.); wojciech.zwierello@pum.edu.pl (W.Ż.); 3 Department of Toxicology, Dairy Technology and Food Storage, West Pomeranian University of Technology in Szczecin, al. Piastów 17, 70-310 Szczecin, Poland

**Keywords:** insulin resistance, polycystic ovary syndrome, hydroxy-eicosatetraenoic acids, biomarkers, polyendocrine metabolic ovarian syndrome

## Abstract

Polyendocrine Metabolic Ovarian Syndrome (PMOS) is one of the most commonly diagnosed endocrine disorders among women of reproductive age. The proposed term more accurately reflects the pathophysiology of the condition, emphasizing its multisystem nature rather than restricting it to ovarian dysfunction. Polycystic ovary syndrome (PCOS) is estimated to affect approximately 6–20% of women of reproductive age. The condition is characterized by considerable clinical heterogeneity, including menstrual irregularities, hyperandrogenism, difficulties conceiving, and abdominal obesity. In addition to hormonal disturbances, an important and often underestimated aspect of PMOS is its association with insulin resistance and chronic low-grade inflammation. Current evidence indicates that insulin resistance represents an important component of the pathogenesis of PMOS. In this context, the identification of biomarkers reflecting metabolic and inflammatory disturbances in affected women is of particular interest. Pro-inflammatory lipid mediators, such as hydroxyeicosatetraenoic acids (HETEs) and hydroxyoctadecadienoic acids (HODEs), are products of fatty acid oxidation and act as active modulators of the inflammatory response. Increased concentrations of these metabolites have been reported in individuals with type 2 diabetes, excessive body weight, cardiovascular diseases, and in women with PMOS. The study included 45 women diagnosed with PMOS, who were divided into groups according to the presence of insulin resistance assessed using the HOMA-IR index. Venous blood plasma was used as the biological material, and the concentrations of selected HETE and HODE metabolites were determined using high-performance liquid chromatography (HPLC). The obtained data were subjected to statistical analysis to assess the associations between lipid mediator concentrations, parameters of carbohydrate metabolism, and selected metabolic indices. Statistical analysis revealed no significant differences in the concentrations of the analyzed oxylipins (HETE and HODE) between patient groups stratified according to HOMA-IR. No significant direct correlations were observed between most of the analyzed metabolites and body mass index (BMI) or fasting blood glucose levels. However, a nominally significant association was identified for 12S-HETE (*p* = 0.0258), which did not remain significant after Bonferroni correction (adjusted *p* = 0.1806). Its concentration was nearly twofold higher in patients with PMOS who exhibited postprandial hyperinsulinemia, assessed two hours after an oral glucose tolerance test (OGTT), compared with women who had a normal insulin response. Among the seven oxylipin metabolites analyzed, only 12S-HETE showed a nominal association with postprandial hyperinsulinemia. At the same time, the findings suggest that HOMA-IR and glycemic parameters may not fully reflect changes in the oxylipin profile in patients during the early stages of metabolic dysfunction. The concentrations of several analyzed oxylipins were strongly correlated, whereas 16RS-HETE showed only weak correlations with the other metabolites. Further studies are needed to clarify the relationship between oxylipin concentrations and metabolic dysfunction in women with PCOS/PMOS.

## 1. Introduction

The pathophysiology of polycystic ovary syndrome/polyendocrine metabolic ovarian syndrome (PCOS/PMOS) is complex and multifactorial. Hormonal and metabolic disturbances, together with genetic and environmental factors, contribute to the development and course of the disorder. The clinical presentation remains highly heterogeneous; therefore, the proposed nomenclature, PMOS, may better reflect the systemic nature of the disease. Hormonal disturbances, particularly hyperandrogenism, together with insulin resistance and associated metabolic abnormalities, play a key role in the pathomechanisms of the disorder [1]. Endocrine abnormalities in PMOS are primarily related to impaired gonadotropin secretion by the pituitary gland [2]. Insulin resistance occurs in approximately 60–70% of women with PMOS, irrespective of body weight [3]. It is characterized by reduced sensitivity of peripheral tissues, including skeletal muscle, adipose tissue, and the liver, to insulin, resulting in compensatory hyperinsulinemia. Elevated insulin concentrations contribute to hyperandrogenism by stimulating ovarian androgen production and reducing hepatic synthesis of sex hormone-binding globulin (SHBG), thereby increasing circulating free, biologically active androgens [1]. Metabolic abnormalities associated with PMOS include visceral obesity, dyslipidemia, impaired glucose tolerance, type 2 diabetes mellitus, and arterial hypertension, contributing to an increased risk of metabolic syndrome and cardiovascular disease [3,4].

Current research increasingly emphasizes chronic low-grade inflammation as a mechanism involved in the development and persistence of PMOS. Patients with PMOS exhibit elevated concentrations of pro-inflammatory cytokines, including TNF-α and IL-6, increased C-reactive protein (CRP), and enhanced oxidative stress. Lipid peroxidation products, such as hydroxyeicosatetraenoic acids (HETEs) and hydroxyoctadecadienoic acids (HODEs), are considered important inflammatory mediators involved in the pathophysiology of the syndrome [4]. These lipid mediators are generated through the oxidation of unsaturated fatty acids and participate in inflammatory responses, oxidative stress, cell adhesion, and intercellular communication. HETEs are metabolites of arachidonic acid (AA), a component of cell membrane phospholipids [5]. Different HETE isoforms, including 5-HETE, 12-HETE, and 15-HETE, are generated by lipoxygenases (LOX), depending on the position of AA hydroxylation. Their biological effects differ: 5-HETE participates in leukotriene synthesis, 12-HETE exhibits chemotactic activity and may promote monocyte adhesion to the vascular endothelium, whereas 15-HETE exerts context-dependent effects, including anti-inflammatory and pro-inflammatory activities [6]. HODEs are derived from linoleic acid (LA), with 9-HODE and 13-HODE being the main forms. They are generated enzymatically, mainly through lipoxygenase (LOX) pathways, as well as by non-enzymatic lipid peroxidation induced by reactive oxygen species. HODEs act as ligands for nuclear receptors, including PPAR-γ, thereby influencing genes involved in lipid and glucose metabolism and inflammatory responses [7]. Experimental data indicate that 9-HODE and 13-HODE may interfere with insulin signalling through the insulin receptor (IR) and insulin receptor substrate (IRS) proteins, impairing glucose transport into insulin-dependent tissues and contributing to reduced insulin sensitivity [8]. Consequently, HETEs and HODEs have been proposed not only as indicators of chronic low-grade inflammation and metabolic disturbances but also as potential biomarkers of insulin resistance and therapeutic targets in PMOS [9]. Li et al. reported significantly higher 13-HODE concentrations in women with PMOS than in controls, with levels correlating with insulin concentrations and BMI [10]. Increased 12-HETE concentrations have also been observed in patients with PMOS irrespective of obesity. In addition, 13-HODE may inhibit phosphorylation of the insulin receptor and IRS-1, further impairing glucose transport and insulin sensitivity [11]. These metabolites also influence immune function, energy metabolism, and intercellular communication [12]. Long-term metformin treatment has been associated with reduced HETE and HODE concentrations, suggesting a relationship between these metabolites, insulin resistance, and inflammation [13]. Identification of sensitive and specific biomarkers could improve diagnosis, metabolic phenotyping, and therapeutic decision-making [14]. Advances in analytical technologies and lipidomics may further support the incorporation of HETEs and HODEs into expanded PMOS diagnostics, particularly for metabolic abnormalities and the early identification of complications such as type 2 diabetes, arterial hypertension, and cardiovascular disease [15].

Environmental factors, including dietary habits, physical activity, and exposure to environmental toxins, may contribute to oxidative stress. Oxidative stress can promote lipid peroxidation and the formation of oxidized lipid products, including HODEs and certain HETE forms. Oxidative stress has been linked to reproductive and metabolic disturbances, including insulin resistance, in women with PCOS [16]. Genetic polymorphisms, including variants in the gene encoding peroxisome proliferator-activated receptor gamma (PPAR-γ), have been investigated in women with PCOS. PPAR-γ regulates genes involved in lipid and carbohydrate metabolism and contributes to suppression of inflammatory responses. The Pro12Ala polymorphism has been associated with PCOS susceptibility and selected metabolic parameters, although its relationship with insulin resistance remains unclear [17].

The study aimed to assess whether the analyzed mediators could serve as potential biomarkers of metabolic disturbances and chronic low-grade inflammation associated with PMOS. An additional objective was to identify oxylipins associated with early metabolic dysfunction in women with PMOS and evaluate their potential as biomarkers of postprandial hyperinsulinemia.

## 2. Results

### 2.1. HETE and HODE Concentrations

Seven oxylipin metabolites were collected and analyzed. None of the metabolites showed a normal distribution; all exhibited right-skewed distributions. Most patients had low or moderate concentrations, whereas disproportionately high concentrations were observed in some patients. The results, together with the arithmetic mean concentrations in the study group, are presented in Table 1.

Considering the lack of normal distribution, the minimum, maximum, and median values of the individual parameters are presented in Table 2.

Next, Spearman correlation coefficients were calculated to assess the relationships between the analyzed HETE and HODE metabolites. The results are presented in Table 3.

The data presented in Table 3 indicate that the strongest correlations (>0.8) were observed between the following metabolites:

- 15S HETE and 5 HETE showed a very strong correlation (0.909);
- 15S HETE and 12S HETE showed a strong correlation (0.841).

5 HETE and 5 Oxo Ete Showed A Strong Correlation (0.888):

- 13S HODE and 5 HETE (0.831);
- 12S HETE and 5 oxo ETE showed a strong correlation (0.846).

Other Positive Correlations Were Observed Between:

- 13S HODE and 15S HETE (0.777);
- 13S HODE and 5 oxo ETE (0.797);
- 9S HODE and 5 HETE (0.642);
- 9S HODE and 5 oxo ETE (0.665).

16RS HETE showed only weak correlations with the other analyzed metabolites.

### 2.2. Insulin Resistance Indices

As with the HETE/HODE metabolites, the insulin resistance indices were assessed for normality of distribution. All analyzed parameters showed distributions that significantly deviated from normality (*p* < 0.05 for each parameter). These findings justified the use of non-parametric tests in subsequent statistical analyses involving these variables.

The median, minimum and maximum values, lower and upper quartiles, and interquartile ranges were then determined. The results are presented in Table 4.

Spearman correlation coefficients were used to assess the relationships between the individual parameters. The results are presented in Table 5.

Spearman correlation coefficients are presented above the diagonal, and the corresponding *p*-values are presented below the diagonal.

The strongest correlation in the entire matrix was observed between fasting insulin and HOMA-IR (ρ = 0.963; *p* < 0.0001). A strong positive correlation was also found between 2-h glucose and 2-h insulin concentrations (ρ = 0.618; *p* < 0.0001).

Significant, Although Moderate, Correlations Were Also Observed Between:

- Fasting insulin and 2-h insulin (ρ = 0.523; *p* = 0.0005);
- Fasting glucose and 2-h glucose (ρ = 0.498; *p* = 0.0011).

These findings indicate a degree of consistency in the glucose and insulin profiles of individual patients. Patients with higher fasting values tended to also have higher values following the glucose load.

HOMA-IR showed significant correlations with all analyzed parameters. In addition to the very strong association with fasting insulin, HOMA-IR was moderately correlated with 2-h insulin (ρ = 0.564; *p* = 0.0002) and fasting glucose (ρ = 0.454; *p* = 0.0017), and more weakly with 2-h glucose (ρ = 0.376; *p* = 0.0169).

### 2.3. Correlation Analysis Between HETE/HODE Metabolites and Insulin Resistance

Table 6 presents Spearman correlation coefficients between the concentrations of seven oxylipin metabolites and parameters characterizing carbohydrate metabolism and insulin resistance. This analysis represents a key component of the study, addressing the relationship between oxylipins and insulin resistance in PMOS. All correlation coefficients ranged from −0.240 to +0.430, indicating weak or, at most, moderate associations between the analyzed variables (Table 6).

To determine whether oxylipin metabolite concentrations differed between insulin-resistant and insulin-sensitive patients, the Mann–Whitney U test was performed.

Patients were divided into two groups according to HOMA-IR, using a threshold of 2.5. This resulted in group A, comprising insulin-resistant patients (HOMA-IR > 2.5; *n* = 24), and group B, comprising insulin-sensitive patients (HOMA-IR ≤ 2.5; *n* = 21). The results are presented in Table 7.

The analysis revealed no statistically significant differences in the concentrations of any of the seven analyzed metabolites between the two groups. The median concentration of 16RS HETE was lower in the insulin-resistant group (0.024) than in the insulin-sensitive group (0.045); however, this difference did not reach statistical significance (U = 204.0; *p* = 0.2611). Similarly, the concentrations of the remaining metabolites were comparable between the groups (Table 7).

Patients were subsequently divided into two groups according to 2-h insulin concentration following the glucose load, using a threshold of 60 µIU/mL. One group comprised patients with a normal insulin response, defined as 2-h insulin ≤ 60 µIU/mL, whereas the second group comprised patients with postprandial hyperinsulinemia, defined as 2-h insulin > 60 µIU/mL. The results of the Mann–Whitney U test are presented in Table 8.

A nominally significant difference in 12S HETE concentration was observed between the study groups (U = 117; *p* = 0.0258), but it did not remain significant after Bonferroni correction (adjusted *p* = 0.1806).

The median concentration of this metabolite was nearly twofold higher in patients with postprandial hyperinsulinemia (1.337) compared with patients with a normal insulin response (0.709). This finding is consistent with the previously observed correlation between 12S HETE and 2-h insulin concentration and may indicate a potential association between this metabolite and the dynamic insulin response to a glucose load.

The remaining metabolites did not show statistically significant differences between the groups (Table 8).

Finally, patients were divided into two groups according to their 2-h glucose concentration following the glucose load during the OGTT, using a threshold of 100 mg/dL. The results are summarized in Table 9. The analysis revealed no statistically significant differences in the concentrations of any of the seven oxylipin metabolites between the groups. Metabolite concentrations were comparable between the two groups, with no statistically significant differences observed (Table 9).

Comparison of the insulin-resistant and insulin-sensitive groups using the Mann–Whitney U test confirmed these observations. Despite significant differences between the groups in anthropometric parameters, including body weight, fat mass, waist circumference, and hip circumference, as well as insulin-related parameters, including fasting and post-load insulin concentrations, no statistically significant differences were observed in the concentrations of any of the seven analyzed oxylipin metabolites (all *p* > 0.05). Different results were obtained when the relationship between oxylipins and postprandial insulinemia was analyzed. In the correlation analysis, the strongest association was observed between 12S HETE and 2-h insulin concentration. Positive, although weaker, correlations with postprandial insulin were also observed for 5 oxo ETE, 13S HODE, 15S HETE, 5 HETE, and 9S HODE.

Patients with postprandial hyperinsulinemia had nominally significantly higher concentrations of 12S HETE compared with patients with a normal insulin response, although this difference did not remain significant after correction for multiple comparisons.

For 5 oxo ETE, the median concentration was more than twice as high in the hyperinsulinemic group, although the difference was not statistically significant. In contrast to postprandial insulinemia, postprandial glycemia was not associated with the analyzed oxylipin metabolites. Comparison of the groups according to 2-h glucose concentration using the Mann–Whitney U test revealed no significant differences for any of the metabolites.

Overall, the study suggests a potential association between 12S HETE and postprandial hyperinsulinemia, but not with insulin resistance assessed using HOMA-IR or with glycemia. This observation requires confirmation in further studies.

An additional exploratory regression analysis adjusting for BMI showed a positive association between 12S-HETE and 2-h insulin (B = 27.72, *p* = 0.0291; *n* = 40). However, the association was not statistically significant after excluding observations recorded as zero for 12S-HETE (B = 22.49, *p* = 0.179; *n* = 32).

## 3. Discussion

The analysis identified an exploratory association between 12S-HETE concentration and postprandial hyperinsulinemia, defined as the insulin concentration measured two hours after the oral glucose tolerance test. This association was observed in the absence of statistically significant correlations with fasting glycemia or the HOMA-IR index. This finding is of clinical interest, as 53.3% of the study population met the criteria for insulin resistance (HOMA-IR > 2.5), whereas only 13.3% had impaired fasting glycemia. This observation appears to differ from some previous studies linking increased concentrations of metabolites from the 12-lipoxygenase pathway with insulin resistance. Previous studies have shown that 12S-HETE is positively and significantly correlated with HOMA-IR, the hepatic steatosis index, and adipokines such as leptin in young adults with clinically manifest metabolic syndrome [18].

However, the present findings may reflect differences in metabolic responses among women with PCOS/PMOS, although pancreatic β-cell function was not directly assessed [18,19]. Recent evidence suggests that chronic hyperinsulinemia, particularly during the postprandial phase, may represent not only a consequence but also an early driver of insulin resistance, making it a more sensitive indicator of acute metabolic stress [19]. Thus, the association of 12S-HETE with dynamic rather than static metabolic parameters is consistent with evidence that lipoxygenase activity may respond to acute glucose-induced stress in pancreatic β-cells [20]. The present findings are also consistent with reports indicating that insulin resistance in PMOS does not necessarily manifest initially as elevated blood glucose but may instead be accompanied by compensatory hyperinsulinemia. Similar relationships have been described by Zhao et al. [1] and Amisi [3], who emphasized that hyperinsulinemia may precede overt disturbances in carbohydrate metabolism. Therefore, the absence of correlations between oxylipins and glycemia does not exclude their involvement in metabolic dysfunction, although the stage of these abnormalities cannot be determined from the present study.

In experimental settings, increased secretory activity and functional adaptation of pancreatic islets may be associated with enhanced arachidonic acid turnover in cell membranes, potentially resulting in increased production of 12S-HETE [21]. Elevated circulating insulin concentrations may also stimulate arachidonic acid metabolism and lipoxygenase activity in other tissues, contributing to increased circulating 12S-HETE [22]. The present findings are partly consistent with observations by Jurado-Fasoli et al., who reported an association between omega-6 oxylipins and cardiometabolic risk [18]. However, in the present study, the strongest associations were observed with the insulin response following glucose administration rather than with HOMA-IR, suggesting a potential relationship between 12S-HETE and the dynamic insulin response. Another finding concerns 5-oxo-ETE. In the study population, this metabolite showed a statistically non-significant trend toward differentiating patients according to postprandial hyperinsulinemia, with a median concentration more than twice as high in women whose 2-h insulin concentration exceeded 60 µIU/mL. Recent clinical studies have confirmed the presence and potential significance of 5-oxo-ETE in plasma from patients with systemic inflammatory conditions, including metabolic syndrome, obesity, and pregnancies complicated by gestational diabetes mellitus [23]. Systemic activation of inflammatory pathways was also reflected in the Spearman correlation matrix, which identified a group of strongly correlated metabolites (ρ > 0.8), comprising 15S-HETE, 12S-HETE, 5-HETE, and 5-oxo-ETE. This profile is consistent with the low-grade chronic inflammatory state described in obesity, prediabetes, and type 2 diabetes mellitus [24].

A different pattern was observed for the linoleic acid-derived metabolites 13S-HODE and 9S-HODE. Although both showed weak or moderate positive correlations with insulin concentrations following glucose administration, these associations were considerably weaker than those observed for arachidonic acid-derived metabolites, and the Mann–Whitney U tests did not demonstrate their usefulness in differentiating the metabolic groups. HODEs have been investigated as biomarkers of systemic oxidative stress and indicators of lipid peroxidation, with increased concentrations reported, among other conditions, in women with polycystic ovary syndrome. The predominance of HETE rather than HODE metabolites in this early-risk population may indicate a greater contribution of specific arachidonic acid metabolic pathways. This interpretation is supported by studies showing that, in diabetes and the early stages of impaired glucose tolerance, the most pronounced changes involve the highly enzymatic 12/15-LOX pathways, which preferentially utilize AA as a substrate [25]. Previous studies by Szczuko et al. [4] demonstrated the involvement of linoleic acid- and arachidonic acid-derived metabolites in the inflammatory and metabolic profile of women with PMOS. The present results extend these observations by indicating that the analyzed lipid mediators do not have equivalent interpretative value.

**Figure 1:**
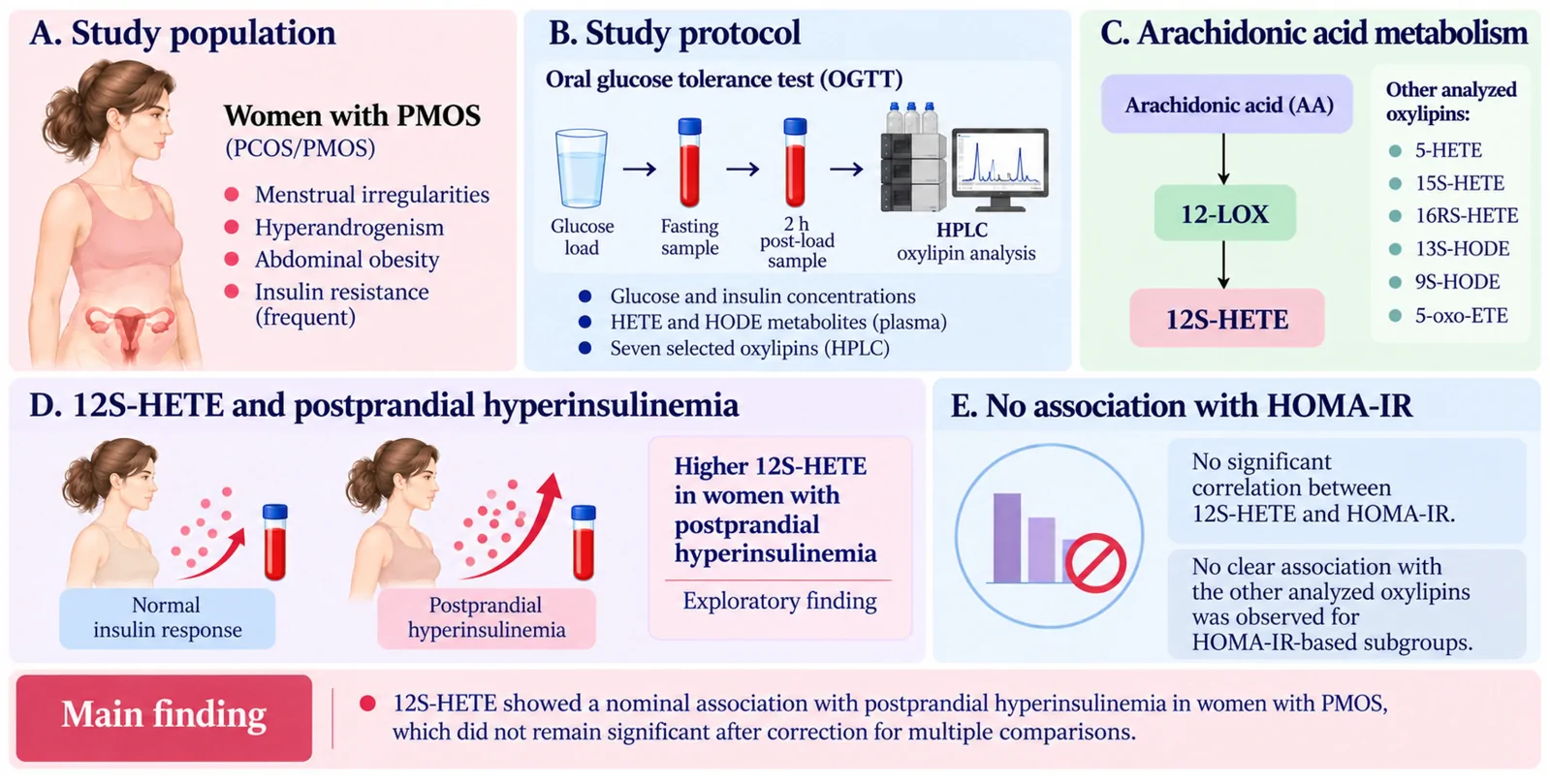
Study protocol and main findings on arachidonic acid metabolism in women with PCOS/PMOS, including the association between 12S-HETE and postprandial hyperinsulinemia.

The metabolite 16RS-HETE did not show a significant correlation with any of the analyzed parameters and did not differentiate insulin-sensitive from insulin-resistant patients. In contrast to the other oxylipins examined, 16RS-HETE is not generated through the lipoxygenase pathway but results from ω-hydroxylation of arachidonic acid catalyzed by cytochrome P450 isoenzymes. The absence of differences in 16RS-HETE concentrations between insulin-resistant and insulin-sensitive patients is consistent with the possibility that early insulin resistance is associated predominantly with changes in LOX-dependent pathways in insulin-responsive tissues. This may represent a form of metabolic selectivity, as described in studies examining differences in the mechanisms responsible for the production of bioactive lipids [26]. Among the metabolites analyzed, 12S-HETE showed the most distinct nominal association, although preferential activation of the 12-lipoxygenase pathway cannot be confirmed from the present data. This interpretation is supported by studies addressing the role of 12-lipoxygenase in pancreatic β-cell function. Tersey et al. [25] and Ma et al. [27] reported that products of the 12-LOX pathway may affect β-cell viability and their ability to maintain appropriate insulin secretion. However, the present study did not directly assess pancreatic β-cell function or the tissue origin of circulating 12S-HETE. The results discussed below are presented in Figure 1.

The interpretation of the obtained results should take into account several important methodological and clinical limitations. One of the main limitations is the sample size: the study included 45 patients, which represents a relatively small cohort for correlation analyses and subgroup comparisons. With this sample size, the statistical power to detect weak and moderate effects (ρ < 0.4) is limited. The association between 12S-HETE and 2-h insulin did not remain significant after correction for multiple comparisons. The cross-sectional design does not allow causal conclusions, and pancreatic β-cell function or tissue-specific insulin resistance was not directly assessed. Furthermore, age was not available for adjustment, and the results may be affected by other potential confounding factors. The regression results were sensitive to the handling of zero values recorded for 12S-HETE, whose analytical status could not be confirmed.

In light of the present findings, it would be particularly valuable to measure 12S-HETE together with insulin and glucose at subsequent time points, for example at 0, 60, and 120 min. Such an approach would allow assessment of whether the increase in 12S-HETE precedes hyperinsulinemia, occurs concurrently with it, or represents a consequence of the insulin response. The use of these mediators in clinical practice is also subject to certain limitations. In particular, established reference ranges for individual HETE and HODE forms are lacking both in the general population and among women with PMOS. Furthermore, analytical methods used for their determination such as HPLC-DAD or those coupled with tandem mass spectrometry (LC-MS/MS), although highly precise, are not yet widely available in routine clinical practice.

## 4. Materials and Methods

### 4.1. Characteristics of the Study Group

The study included 45 women diagnosed with PMOS according to the Rotterdam criteria [28]. All women met at least two of the three diagnostic criteria: clinical or biochemical signs of hyperandrogenism, oligo-ovulation or anovulation, and polycystic ovarian morphology on ultrasound (Ultrasound Voluson 730, GE, Zipf, Switzerland). The study group consisted of non-pregnant women aged 18–45 years who either presented to the outpatient clinic because of difficulties conceiving or continued previously initiated treatment. The exclusion criteria included age <18 years and other conditions that may present with similar symptoms (ovarian or adrenal tumors that secrete androgens, primary ovarian failure, Cushing syndrome, and thyroid dysfunction). According to body mass index (BMI), 18 women were obese (BMI ≥ 30), 16 were overweight (BMI 25–29.9), and 11 had normal body weight (BMI 18.5–24.9). The biochemical and glycemic parameters of the study group are presented in Table 10A.

Women were subsequently divided into two groups according to the presence of insulin resistance (Table 10B). Group A (IR+) comprised patients with insulin resistance, defined as HOMA-IR > 2.5 (*n* = 24), whereas Group B (IR−) comprised insulin-sensitive patients with HOMA-IR within the reference range (*n* = 21). To minimize factors that could potentially affect lipid measurements and the assessment of insulin resistance, exclusion criteria were also defined. Women receiving medications known to affect carbohydrate or lipid metabolism were excluded [29].

These included:

- Oral contraceptives and other hormonal preparations, due to their effects on SHBG, androgen, and insulin concentrations;
- Metformin and other glucose-lowering medications, which directly improve insulin sensitivity and could therefore interfere with the assessment of endogenous insulin resistance;
- Statins and fibrates, which modify the lipid profile;
- Non-steroidal anti-inflammatory drugs (NSAIDs) and glucocorticoids, which may affect eicosanoid pathways and alter the concentrations of inflammatory mediators.

Patients with chronic conditions that could independently affect the parameters under investigation were also excluded. Type 1 or type 2 diabetes mellitus was considered an exclusion criterion. Women with significant cardiovascular diseases, such as uncontrolled arterial hypertension or ischemic heart disease, as well as those with severe hepatic or renal diseases, including liver cirrhosis or chronic kidney failure, were excluded because of the potential effects of these conditions on lipid and drug metabolism. Women who reported alcohol abuse or heavy smoking were also excluded, as alcohol consumption and tobacco smoke may alter lipid metabolism and increase inflammatory markers. The exclusion criteria were established to obtain a study population in which the observed insulin-related abnormalities could be attributed primarily to PMOS rather than to comorbidities and/or pharmacological treatment.

### 4.2. Biological Material

Venous blood samples were collected using a standard venipuncture procedure from the antecubital vein. The samples were stored at 4 °C for a maximum of 30 min before centrifugation at 3000× *g* for 10 min at 4 °C. Following centrifugation, the plasma was collected and transferred into Eppendorf tubes. The plasma samples were subsequently frozen at −80 °C and stored until analysis [30].

### 4.3. Biochemical Analysis by HPLC

The HPLC separations were performed using an Agilent Technologies 1260 liquid chromatograph Agilent Technologies, Cheadle, UK), consisting of a degasser (model G1379B), bin pump (model G1312B), column oven (model G1316A) and diode array detector (model G1315C DAD VL+). HETE and HODE derivatives were isolated from plasma samples using solid-phase extraction. Proteins and other unwanted substances were retained on the sorbent, while the analytes in the filtrate were subsequently separated on a C18 column (RP-18 SPE columns, Agilent Technologies, Cheadle, UK) [20]. A solvent gradient was applied, involving a gradual increase in the proportion of methanol in the mobile phase, which enabled the sequential elution of HETE and HODE isomers based on differences in polarity and the strength of their interactions with the stationary phase. Each isomer (5-HETE, 12-HETE, 15-HETE, 13S-HODE, and 9S-HODE) exhibited a characteristic retention time, allowing individual separation and identification on the chromatogram. UV detection was performed at a wavelength of 235 nm for 5-HETE, 12-HETE, 15-HETE, 9S-HODE, and 13S-HODE, whereas a wavelength of 280 nm was used specifically for 5-oxo-ETE, whose ketone group results in a different absorption maximum [31]. UV detection enabled simultaneous quantification of all analyzed mediators within a single chromatographic run, without the need for repeated measurements [4]. Quantitative analysis was conducted using ChemStation Software (LTS 01.11) (Agilent Technologies, Cheadle, UK).

### 4.4. Methods for Assessing Insulin Resistance

A detailed assessment of glucose and insulin metabolism was performed in women with PMOS using standardized methods. Two complementary approaches were applied:

1. The homeostatic model assessment of insulin resistance (HOMA-IR), based on fasting glucose and insulin concentrations;
2. The oral glucose tolerance test (OGTT), used to assess the metabolic response to a glucose load.

The HOMA-IR index was calculated based on fasting glucose and insulin concentrations [32]. Patients were classified as insulin-resistant when HOMA-IR exceeded 2.5. HOMA-IR primarily reflects hepatic insulin sensitivity through the regulation of fasting glucose levels, whereas it may not adequately capture abnormalities in peripheral glucose utilization, particularly in skeletal muscle, that become apparent only following a glucose challenge [33].

The OGTT was performed according to WHO/ADA recommendations. Following an overnight fast of at least 8–12 h, in the morning, participants received 75 g of anhydrous glucose dissolved in 250 mL of water orally. Venous blood glucose concentrations were measured immediately before glucose administration and 120 min after the glucose load. Insulin concentrations were determined at the same time points, allowing assessment of the post-load insulin response according to the adopted reference ranges [34]. A normal 2-h glucose concentration was defined as <7.8 mmol/L (<140 mg/dL); impaired glucose tolerance (IGT) was defined as 7.8–11.0 mmol/L (140–199 mg/dL), whereas diabetes was defined as ≥11.1 mmol/L (≥200 mg/dL). Some analyses involving OGTT-derived variables included fewer participants because of missing measurements. In healthy individuals, blood glucose levels return to normal within two hours due to effective insulin secretion and adequate peripheral tissue sensitivity to insulin. In individuals with impaired glucose tolerance, excessive elevation or persistence of elevated glucose concentrations may be observed, often accompanied by hyperinsulinemia as a marker of compensatory insulin secretion [35].

### 4.5. Statistical Analysis

The statistical analysis was adapted to the characteristics of the data and the size of the study population. Statistical analysis was performed with Statistica 12.0 (Statsoft, Tulsa, OK, USA). The Shapiro–Wilk test was used to assess the normality of data distribution, as recommended for smaller groups of patients in medical research. For parameters showing significant deviations from a normal distribution (*p* < 0.05), including the analyzed variables, median values were determined. Subsequently, the Mann–Whitney U test was applied to compare two independent groups [36]. The strength and direction of monotonic associations between pairs of variables were assessed using Spearman’s rank correlation coefficient. The analysis focused on the associations between concentrations of the lipid inflammatory mediators HETE and HODE and parameters of glucose and insulin metabolism. Spearman’s rank correlation was selected because the distributions of the analyzed variables deviated from normality and because of the potential presence of outlying observations.

Statistical significance was defined as *p* < 0.05. The reported analyses comprised 21 correlations between oxylipins, 10 correlations between metabolic parameters, 35 correlations between oxylipins and metabolic parameters, and 21 subgroup comparisons. For the seven oxylipin comparisons according to 2-h insulin concentration (Table 8), Bonferroni-adjusted *p*-values were calculated to account for multiple testing. The other correlation and subgroup analyses were considered exploratory. An additional exploratory multiple linear regression analysis was performed to assess the association between 12S-HETE and 2-h insulin concentration after adjustment for BMI. A sensitivity analysis was performed excluding observations recorded as zero for 12S-HETE. Correlation coefficients were interpreted according to both statistical significance and the strength of the association. Correlation coefficients of approximately 0.1–0.3 were considered indicative of a weak correlation, values of 0.3–0.5 as moderate, and values > 0.5 as strong.

## 5. Conclusions

Among the seven oxylipin metabolites analyzed, only 12S-HETE showed a nominal association with postprandial hyperinsulinemia. Glycemic parameters, both fasting and following a glucose challenge, were not clearly associated with changes in the oxylipin profile in the study population. The present study does not allow conclusions regarding the temporal relationship between tissue insulin resistance and glycemic abnormalities. Insulin measured following a glucose challenge showed a stronger association with inflammatory biomarkers than the static HOMA-IR index. The observed differences in oxylipin concentrations may be related to variations in the insulin response following a glucose challenge. The present study indicates a possible exploratory association between 12S-HETE and the dynamic insulin response to a glucose challenge, in the absence of a similar relationship with fasting insulin resistance or glycemia.

## Figures and Tables

**Table 1:** Arithmetic mean concentrations of HETE and HODE metabolites in the study group.

Metabolites (ng/mL)	Mean	Shapiro–Wilk W	Shapiro–Wilk *p*
16RS HETE	0.0346	0.8321	0.0000
15S HETE	0.5855	0.8260	0.0000
12S HETE	1.0273	0.7841	0.0000
5 HETE	0.2942	0.9243	0.0059
13S HODE	0.1124	0.9227	0.0052
9S HODE	0.1557	0.9217	0.0048
5 oxo ETE	0.1675	0.7795	0.0000

**Table 2:** Medians and interquartile ranges of HETE and HODE metabolites.

Metabolites (ng/mL)	Median	Min.	Max.	Q1 (25%)	Q3 (75%)	IQR (Q3–Q1)
16RS HETE	0.0371	0.0000	0.1480	0.0000	0.0536	0.0536
15S HETE	0.5300	0.0403	2.4365	0.2315	0.7286	0.4970
12S HETE	0.9962	0.0000	4.9052	0.2017	1.3425	1.1408
5 HETE	0.2966	0.0000	1.0092	0.0322	0.4329	0.4007
13S HODE	0.0953	0.0000	0.3208	0.0534	0.1742	0.1208
9S HODE	0.1333	0.0121	0.4373	0.0583	0.2337	0.1754
5 oxo ETE	0.0849	0.0000	0.8834	0.0000	0.2504	0.2504

**Table 3:** Spearman correlation coefficients between HETE and HODE metabolites.

Metabolites	16RS HETE	15S HETE	12S HETE	5 HETE	13S HODE	9S HODE	5 Oxo ETE
16RS HETE	1.000	0.161	0.170	0.117	0.167	−0.006	0.105
15S HETE	0.161	1.000	0.841	0.909	0.777	0.812	0.865
12S HETE	0.170	0.841	1.000	0.782	0.625	0.585	0.846
5 HETE	0.117	0.909	0.782	1.000	0.831	0.707	0.888
13S HODE	0.167	0.777	0.625	0.831	1.000	0.642	0.797
9S HODE	−0.006	0.812	0.585	0.707	0.642	1.000	0.665
5 oxo ETE	0.105	0.865	0.846	0.888	0.797	0.665	1.000

**Table 4:** Medians and interquartile ranges of insulin resistance indices.

Parameters	Median	Min.	Max.	Q1 (25%)	Q3 (75%)	IQR (Q3–Q1)
Fasting Insulin	11.8	3.5	37.4	7.00	16.00	9.00
Insulin after 2 h	56.5	7.2	383.9	31.70	99.70	68.00
Fasting glucose	91.5	66.5	132.0	84.91	96.80	11.89
Glucose after 2 h	103.8	56.0	219.3	92.97	129.18	36.20
HOMA-IR	2.8	0.8	8.4	1.58	3.77	2.19

**Table 5:** Spearman correlation coefficients between insulin resistance indices.

Parameters	Fasting Insulin	Insulin After 2 h	Fasting Glucose	Glucose After 2 h	HOMA-IR
Fasting Insulin	1.000	0.523	0.247	0.259	0.963
Insulin after 2 h	0.0005	1.000	0.308	0.618	0.564
Fasting glucose	0.1014	0.0530	1.000	0.498	0.454
Glucose after 2 h	0.1060	0.0000	0.0011	1.000	0.376
HOMA-IR	0.0000	0.0002	0.0017	0.0169	1.000

**Table 6:** Spearman correlation coefficients between the concentrations of seven oxylipin metabolites and insulin resistance indices.

Parameters	Fasting Insulin	Insulin After 2 h	Fasting Glucose	Glucose After 2 h	HOMA-IR
16RS HETE	−0.226	−0.120	−0.040	−0.066	−0.240
15S HETE	−0.068	+0.241	+0.046	+0.065	−0.076
12S HETE	+0.018	+0.430	+0.034	+0.131	−0.009
5 HETE	−0.051	+0.225	−0.091	−0.021	−0.105
13S HODE	+0.051	+0.251	−0.038	−0.111	+0.001
9S HODE	+0.025	+0.183	+0.128	+0.029	+0.078
5 oxo ETE	+0.006	+0.351	+0.057	+0.101	−0.018

Values represent Spearman correlation coefficients (ρ).

**Table 7:** Mann–Whitney U test comparing insulin-resistant (group A) and insulin-sensitive (group B) patients.

Metabolites (ng/mL)	Median A (HOMA-IR > 2.5)	Median B (HOMA-IR ≤ 2.5)	U	*p*
16RS HETE	0.024	0.045	204.000	0.2611
15S HETE	0.519	0.530	263.000	0.8112
12S HETE	1.194	0.984	283.000	0.4865
5 HETE	0.318	0.260	256.000	0.9365
13S HODE	0.098	0.080	269.000	0.7074
9S HODE	0.171	0.103	299.000	0.2901
5 oxo ETE	0.105	0.074	273.000	0.6369

**Table 8:** Mann–Whitney U test comparing the group of patients with normal insulin response and postprandial hyperinsulinemia.

Metabolites (ng/mL)	Median (Ins 2 h ≤ 60 µIU/mL)	Median (Ins 2 h > 60 µIU/mL)	U	*p*	Adjusted *p* (Bonferroni)
16RS HETE	0.026	0.037	223	0.5168	1.0000
15S HETE	0.437	0.589	162	0.3163	1.0000
12S HETE	0.709	1.337	117	0.0258	0.1806
5 HETE	0.214	0.319	174	0.4983	1.0000
13S HODE	0.080	0.099	162	0.3163	1.0000
9S HODE	0.128	0.150	165	0.3571	1.0000
5 oxo ETE	0.074	0.165	141	0.1124	0.7868

*p*-values were adjusted using the Bonferroni correction for seven oxylipin comparisons.

**Table 9:** Mann–Whitney U test comparing groups according to glucose concentration 2 h after the load.

Metabolites (ng/mL)	Median (OGTT 2 h < 100 mg/dL)	Median (OGTT 2 h ≥ 100 mg/dL)	U	*p*
16RS HETE	0.026	0.044	209	0.7998
15S HETE	0.489	0.563	185	0.7046
12S HETE	0.996	1.053	172	0.4628
5 HETE	0.250	0.306	201	0.9784
13S HODE	0.102	0.097	225	0.4983
9S HODE	0.133	0.142	200	1.0000
5 oxo ETE	0.081	0.100	186	0.7220

**Table 10:** Characteristics of the study group.

**(A) Biochemical and Anthropometric Parameters**	**Mean**	**SD**
LH (mIU/mL)	8.45	3.63
FSH (mIU/mL)	5.31	1.19
Estradiol (mIU/mL)	43.9	1.95
Testosteron (ng/mL)	7.01	0.87
Androstendion (ng/mL)	4.65	1.84
SHBG (nmol/L)	36.7	18.7
BMI (kg/m^2^)	29.7	6.14
WHR	0.92	0.08
**(B) Insulin resistance indicators in terms of clinical thresholds**	**Number (*n*)**	**Percentage (%)**
Fasting insulin > 12 µIU/mL	22	48.9
Fasting insulin > 25 µIU/mL	2	4.4
HOMA-IR ≤ 2.5	21	46.7
HOMA-IR > 2.5	24	53.3
Fasting glucose ≥ 100 mg/dL (IFG)	6	13.3
Glucose after 2 h ≥ 140 mg/dL	8	20.0
Insulin after 2 h > 60 µIU/mL	19	47.5

Percentages were calculated based on the number of patients with available measurements (*n* = 45 for fasting parameters and *n* = 40 for 2-h OGTT parameters).

## Data Availability

Data available upon request from the corresponding author.

## Abbreviations

The following abbreviations are used in this manuscript:

BMI	Body Mass Index
COX	Cyclooxygenase
CRP	C-reactive protein
FSH	Follicle-stimulating hormone
HETE	Hydroxyeicosatetraenoic acid
HDL	High-density lipoprotein
HODE	Hydroxyoctadecadienoic acid
HOMA-IR	Homeostasis Model Assessment of Insulin Resistance
HPLC	High-Performance Liquid Chromatography
IR	Insulin receptor
LC-MS/MS	Liquid Chromatography–Tandem Mass Spectrometry
LH	Luteinizing hormone
LOX	Lipoxygenase
PCO	Polycystic ovaries
PCOS	Polycystic Ovary Syndrome
PMOS	Polyendocrine Metabolic Ovarian Syndrome
PPAR-γ	Peroxisome proliferator-activated receptor gamma
SHBG	Sex hormone-binding globulin
WHR	Waist-to-hip ratio
